# Supplementary material for: Do beta-amyloid-targeted interventions improve cognition, physical functioning, and overt behaviour of Alzheimer’s Disease (AD) patients: Protocol for meta-analysis of Phase 3 clinical trials both completed and terminated
Source: PLoS One. 2024 Sep 27;19(9):e0307761. doi: 10.1371/journal.pone.0307761 (PMC11432894; doi:10.1371/journal.pone.0307761)
Supplement: Attachment — Submitted filename: Response to Reviewers.docx [file pone.0307761.s002.docx]

Chelsea Stellick

University of Alberta

250 Riverside Drive – Apt 91

New York, NY 10025

[Bedrejo@ualberta.ca](mailto:Bedrejo@ualberta.ca)

January 7, 2024

Editor-in-Chief,

PLOS ONE

Subject: Rebuttal Letter for Manuscript ID PONE-D-23-05935

Dear Editor-in-Chief,

I hope this letter finds you well. We appreciate the thorough review of our manuscript titled "Do pharmacological interventions targeting beta-amyloid improve cognition, physical functioning, and overt behaviour of Alzheimer’s Disease (AD) patients: A protocol for a meta-analysis of Phase 3 clinical trials both completed and terminated," and we thank the reviewers for their insightful comments and suggestions. We have carefully considered each of the reviewer's points and offer our detailed responses below:

**Reviewer:** This manuscript addresses a protocol for a forthcoming meta-analysis of anti-Aβ therapy for Alzheimer's disease. While the results of the meta-analysis are important, it is difficult to judge the significance of publishing the protocol because the method itself of the meta-analysis is not novel and only items are modified.

**Response:** We acknowledge the reviewer's concern regarding the novelty of the methodology. However, we believe that the new clinical trial data available since the latest systematic reviews of anti-Aβ therapy for Alzheimer's disease warrants an updated review. Furthermore, our systematic review is distinctive in its inclusion of all three domains (cognitive outcomes, outcomes of physical functioning and behavior) and all stages of AD, which to our knowledge, has not been previously attempted. This broader approach will provide a more comprehensive understanding of the effectiveness of anti-Aβ therapy across diverse aspects of Alzheimer's disease.

**Reviewer:** Although grouped together as anti-Aβ drugs in the protocol, the mechanisms of drugs that inhibit Aβ production and those that promote Aβ clearance are very different, and I am concerned that the scientific significance of the results may be diminished by grouping them together as "anti-Aβ drugs".

**Response:** We appreciate the reviewer's concern regarding the grouping of different mechanisms of anti-Aβ drugs. We have taken into account the distinction between drugs that inhibit Aβ production and those that promote Aβ clearance. Our systematic review will categorize and analyze data separately for each intervention type to address this concern. The purpose of including both intervention types is to comprehensively explore the Aβ hypothesis and provide insights into their distinct effects on Alzheimer's disease.

**Reviewer:** The preclinical stage is just the clinically asymptomatic stage, which can include A+T+N- and even A+T+N+ as well as A+T-N-.

**Response:** We acknowledge the reviewer's point and have removed the reference to the preclinical stage from the text.

**Reviewer:** "BETA1" is a typo for BACE1 (beta-site amyloid precursor protein cleaving enzyme 1).

**Response:** We apologize for the typographical error and have corrected it to accurately reference BACE1 in the manuscript.

**Reviewer:** Although many have criticized the approval of Aduhelm because of the ambiguity of the decision criteria by the FDA, I guess no one has described it as "it is a step backwards".

**Response:** We understand the reviewer's perspective and have removed the phrase "it is a step backwards" to ensure accuracy and clarity in our manuscript.

**Reviewer:** At the time of submission, Lecanemab was already approved in addition to Aducanumab, so that should also be listed.

**Response:** We appreciate the reviewer's input and will conduct an updated search to include Lecanemab in our analysis, as per the protocol.

**Reviewer:** Since phase III trials of anti-Aβ drugs were not initiated until 2007 or later, it is more likely that the 2011 NIA-AA criteria are used in the trials rather than the older NINCDS-ADRDA criteria.

**Response:** We acknowledge the reviewer's comment and have reviewed our sources more carefully. While the majority of trials may indeed use the 2011 NIA-AA criteria, we have found instances where the older NINCDS-ADRDA criteria were used. We will provide further clarification in the manuscript regarding the criteria used in the trials.

**Reviewer:** CDR-SB is a composite of cognitive function and global function (probably equal to "physical function" in the article), so for those familiar with clinical trials in Alzheimer's disease, it is uncomfortable to put it alongside ADAS-cog. Refer to the FDA guidance for industry published in 2018.

**Response:** We appreciate the reviewer's suggestion and understand the concern. Our intention is to provide a comprehensive overview of outcome measures used in clinical trials. While we acknowledge the difference between CDR-SB and ADAS-cog, we will retain the information to offer a holistic view of the measures employed in Alzheimer's disease trials. This will also allow readers to see which measures are sensitive to the drugs targeting Aβ.

**Reviewer:** Types of outcome measures: “We are not interested in changes in biomarkers (Cerebrospinal fluid (CSF) indications of beta-amyloid 42, Tau, and phospho-tau) therefore, changes in biomarkers are not included in our study.” In focusing pharmacotherapeutic potentials of AD trials, these important biological information is essential to be reviewed. Authors should include these biomarkers in the outcome measures in the meta-analysis.

**Response:** We acknowledge the importance of biomarker data in Alzheimer's disease trials. Considering this, we have revised our approach and will include changes in biomarkers, specifically CSF indications of beta-amyloid 42, Tau, and phospho-tau, in our outcome measures for the meta-analysis.

**Reviewer:** Study selection: “To facilitate the screening process, the team will use Covidence (see www.covidence.org), a web-based tool designed to support systematic review screening and manage references and full-text PDFs. Duplicates will be removed by the Covidence software.” Authors should explain more clearly what type of process will be handled by Covidence staff.

**Response:** We apologize for any confusion and appreciate the reviewer's feedback. To clarify, the screening process will be solely conducted by the authors using the Covidence software. No external staff or assistance will be involved in this process.

**Reviewer:** Data extraction: “Any discrepancies between the two reviewers were resolved by consensus and by consulting a third reviewer.” Authors should describe clearly who will be the candidate of the third reviewer.

**Response:** We appreciate the reviewer's suggestion and will explicitly mention the initials of the third reviewer who will be consulted in case of discrepancies during data extraction.

**Reviewer:** Quantitative synthesis: “We will calculate the I2 to test for heterogeneity for each pooled result. We will pool our results based on each outcome of interest (ADAS-Cog 11, ADAS-Cog 12, ADAS-Cog 13, ADAS-Cog 14, CDR-SB, MMSE, EQ-5D, EQ-5D (Proxy), QoL-AD, ADCS-ADL, and NPI).” It is known that there are some discrepancies of the scores in the versions of NPI, NPI-Q and NPI-NH. Authors should clarify what version of NPI was used in each clinical trial.

**Response:** We acknowledge the reviewer's concern regarding the discrepancies in versions of NPI. In our manuscript, we will clearly specify the version of NPI used in each clinical trial to ensure transparency and accuracy in our analysis.

We thank the reviewers for their valuable feedback, which has undoubtedly improved the quality and rigor of our manuscript. We have carefully addressed each concern and made the necessary revisions to ensure the accuracy, clarity, and scientific merit of our work. We hope these clarifications and amendments

Thank you,

Chelsea
