# Supplementary material for: Do beta-amyloid-targeted interventions improve cognition, physical functioning, and overt behaviour of Alzheimer’s Disease (AD) patients: Protocol for meta-analysis of Phase 3 clinical trials both completed and terminated
Source: PLoS One. 2024 Sep 27;19(9):e0307761. doi: 10.1371/journal.pone.0307761 (PMC11432894; doi:10.1371/journal.pone.0307761)
Supplement: Attachment — Submitted filename: Response to Reviewers -06302024.docx [file pone.0307761.s003.docx]

Chelsea Stellick

University of Alberta

250 Riverside Drive – Apt 91

New York, NY 10025

[Bedrejo@ualberta.ca](mailto:Bedrejo@ualberta.ca)

June 30, 2024

Editor-in-Chief,

PLOS ONE

Subject: Rebuttal Letter for Manuscript ID PONE-D-23-05935

Dear Editor-in-Chief,

I hope this letter finds you well. We appreciate the thorough review of our manuscript titled "Do pharmacological interventions targeting beta-amyloid improve cognition, physical functioning, and overt behaviour of Alzheimer’s Disease (AD) patients: A protocol for a meta-analysis of Phase 3 clinical trials both completed and terminated," and we thank the reviewers for their insightful comments and suggestions. We have carefully considered each of the reviewer's points and offer our detailed responses below:

**Reviewer:** The author partially complied with the reviewers' comments and revised the manuscript. Let me point out only a few minor points.

1) Please include "Eisai's lecanemab" in P.4, L.9. Since lecanemab is the only drug that is fully approved and already widely used in clinical practice, it is unnatural that it is not mentioned here.

**Response:** We acknowledge the reviewer's point and have added Lecanemab to the text.

**Reviewer:** The author partially complied with the reviewers' comments and revised the manuscript. Let me point out only a few minor points.

2) As another reviewer pointed out, please add "2011 NIA-AA criteria" alongside the NINCDS-ADRDA criteria to the last paragraph of P.6 and inclusion criteria in Table 2. As the author mentioned in response comments, older trials (the number should be larger) used the NINCDS-ADRDA criteria. However, the NINCDS-ADRDA criteria is getting less familiar to inexperienced researchers. This is just to make the paper and its value understandable to more researchers.

**Response:** We acknowledge the reviewer's point and have added 2011 NIA-AA criteria to the text.

**Reviewer:** The author partially complied with the reviewers' comments and revised the manuscript. Let me point out only a few minor points.

3) If it is appropriate, please mention the period during which the paper to be extracted was published. (ex. "published from xx to xx", "published by 2024 xx")

**Response:** We acknowledge the reviewers comment and will include the dates of publication to the systematic review when appropriate

We thank the reviewers for their valuable feedback, which has undoubtedly improved the quality and rigor of our manuscript. We have carefully addressed each concern and made the necessary revisions to ensure the accuracy, clarity, and scientific merit of our work. We hope these clarifications and amendments

Thank you,

Chelsea
